# Data on First Record of Brown Morph Banded Langur (*Presbytis femoralis*), Leucistic Dusky Leaf Monkey (*Trachypithecus obscurus*) in Malaysia and Review of Morph Diversity in Langur (Colobinae)

**DOI:** 10.1016/j.dib.2020.105727

**Published:** 2020-05-21

**Authors:** Mohd Faudzir Najmuddin, Hidayah Haris, Nursyuhada Othman, Fatin Zahari, Abd Rahman Mohd-Ridwan, Badrul Munir Md-Zain, Rohani Shahrool-Anuar, Othman Ayeb, Iqramullah Othman, Muhammad Abu Bakar Abdul-Latiff

**Affiliations:** a Centre of Research for Sustainable Uses of Natural Resources, Faculty of Applied Sciences and Technology, Universiti Tun Hussein Onn Malaysia (Pagoh campus), KM 1, Jalan Panchor, 84600 Muar, Johor, Malaysia; b Centre for Pre-University Studies, Universiti Malaysia Sarawak, 94300 Kota Samarahan, Sarawak, Malaysia; c Department of Biological Sciences and Biotechnology, Faculty of Science and Technology, Universiti Kebangsaan Malaysia, Selangor, 43600 Bangi, Malaysia; d Panz village, Lot 147 Lorong Bahtera, Kg Johor Lama, 81940 Kota Tinggi, Johor, Malaysia; e Rimbawi Geo Discovery, No 109A, Kg Kubang Badak, Mukim Ayer Hangat, 07000 Pulau Langkawi, Malaysia

**Keywords:** Colobinae, Langur, Morph, Albinism, Leucism, Melanin, Malaysia

## Abstract

Morphism refer to polymorphic species, in which multiple colour variants coexist within a population. Morphism in primates is common and langurs also exhibit certain characteristics of morphism, such as conspicuous natal coats. Banded langurs (*Presbytis femoralis*) and dusky leaf monkey (*Trachypithecus obscurus*) exhibits the same characteristics of conspicuous natal coats, but these coats are only limited to infants and changed when they reached adulthood. This article reports the first discovery of rare brown morph of two adult male banded langurs and one leucistic adult female dusky leaf monkey in Malaysia. We also conducted a systematic literature search to review the diversity of morphism in leaf monkey globally.

Specifications table

**Table:** 

Subject	Biology
Specific subject area	Morphology, ecology
Type of data	Figure, table
How data were acquired	Field investigation, Literature search
Data format	Raw
Parameters for data collection	Data collection in Kota Tinggi Johor, and Langkawi Island, Malaysia.
Description of data collection	Scan sampling of ethological observation of *Presbytis femoralis* and *Trachypithecus obscurus* in Malaysia and systematic literature review of colour polymorphism in Colobine.
Data source location	Global coverage
Data accessibility	Data are available with this article

## Value of the data

• This is the first data on brown morph of banded langur (*Presbytis femoralis*) that showed no characteristics of albinism and leucistic dusky leaf monkey (*Trachypithecus obscurus*) in the world.
• The data can be used for primatologist and mammologist especially in the physiological and ecological field to study polymorphism in mammals.
• We recommend that future research should focus on the ecology, behaviour and genetics for both morph of banded langur and dusky leaf monkey to understand their fitness and differences from normal individual.

## 1 Data Description

Polymorphism in primate's hair coloration are not uncommon and different morph in single species have been reported in several literatures [1]. Peculiar morph in langur can be in form of two type; albinism and leucism. Complete albinism refers to the total absence of integumentary and retinal pigmentation [2, 3]. Partial albinism or ‘leucism’ on the other hand were characterized by reduced or absent integumentary pigment, but with pigmented retinas [4]. Other types of morph can be caused by stochastic expression of eumelanin and pheomelanin produced by melanocytes which results in different hair coloration [5]. Here we report of two adult male brown morph banded langur, *Presbytis femoralis* and one leucistic adult female dusky leaf monkey (*Trachypithecus obscurus*) found in Malaysia. The brown morph of banded langur was part of the all-male group which consisted of two brown morph individuals and two normal black morph individuals.

**Fig. 1:**
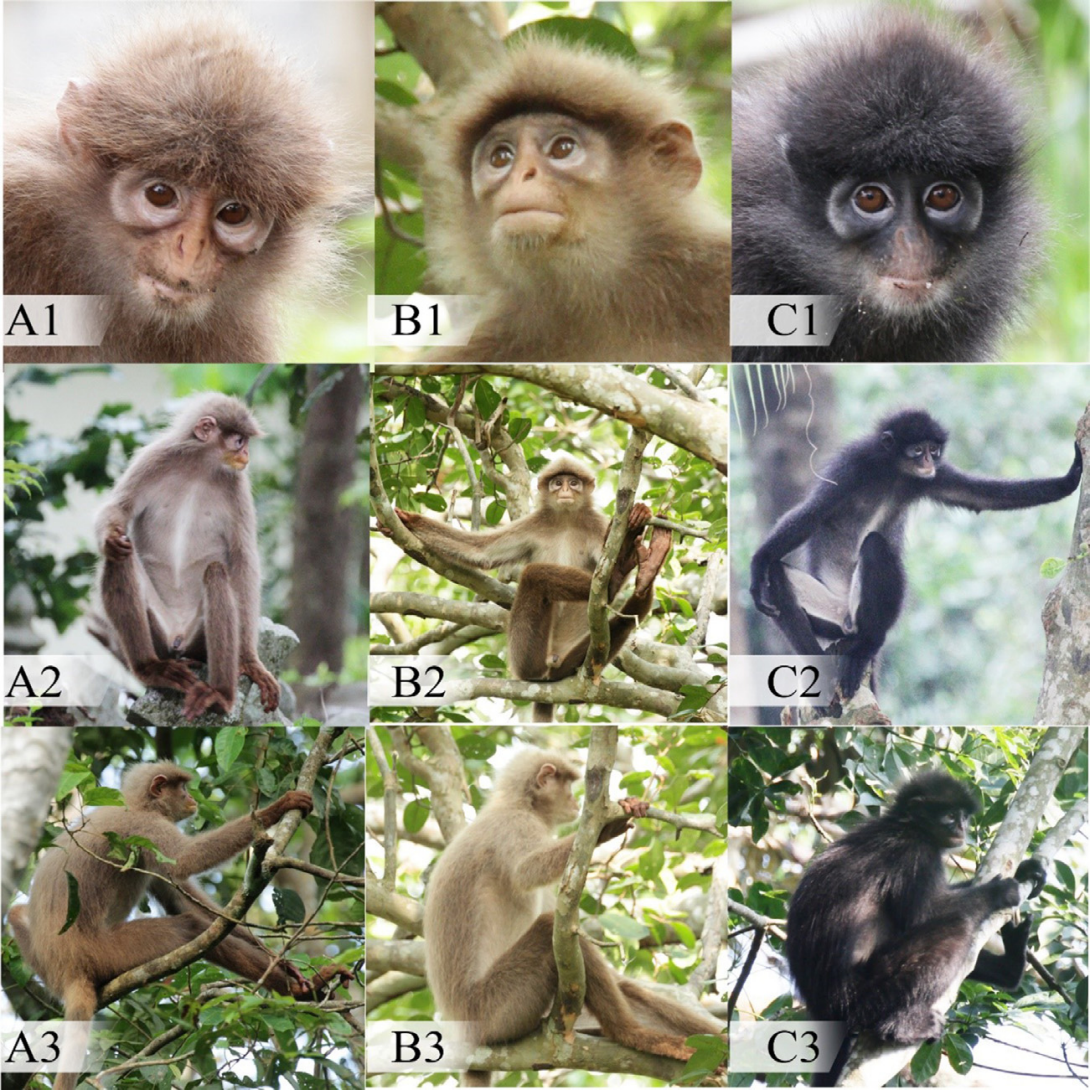
The comparison of pelage coloration between brown morph named Puteh (A1, A2, A3), Dara (B1, B2, B3) and normal morph named Ireng (C1, C2, C3) based on face, ventral and side views.

Fig. 1 illustrates the morphological comparison between Puteh (A), Dara (B) and another black morph individual, Ireng (C). Based on Fig. 1, no congenital red eyes were found in both brown morph individuals, and its pelage coloration was brown in color that neither fit the term leucism nor albinism. The ventral part of both normal and brown morph individuals was almost the same in terms of pelage coloration. From the side views (Fig 1-A3, Fig 1-B3, Fig 1-C3) in Fig. 1, there were no abnormalities in body structure observed for brown morphs, with exception of pelage coloration.

**Fig. 2:**
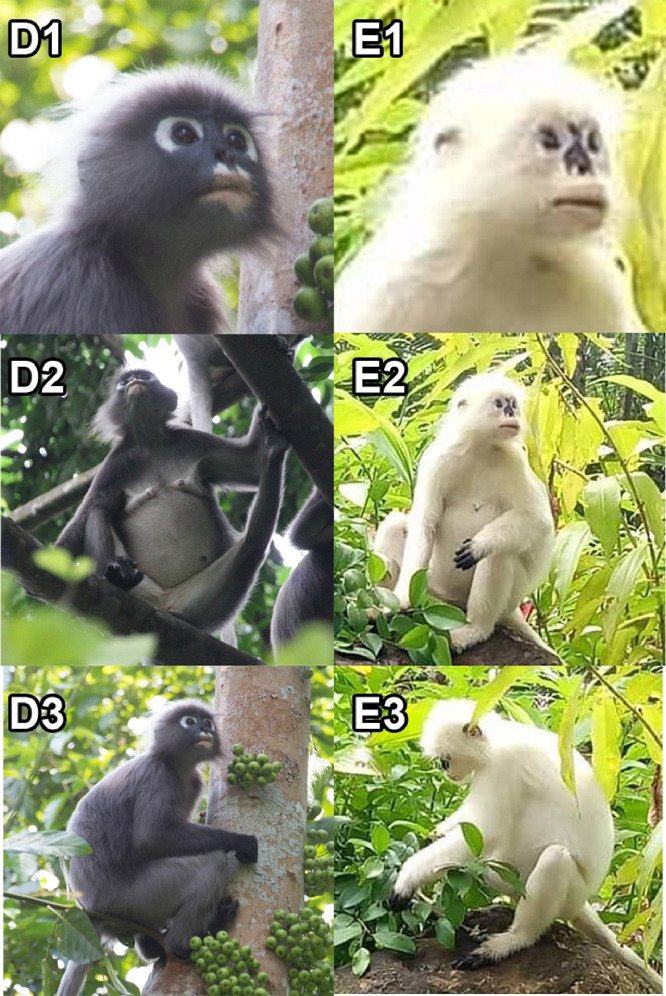
The comparison of pelage coloration between normal morph (D1, D2, D3) and leucistic morph of *T. obscurus* (E1, E2, E3) based on face, ventral and side views.

The white dusky leaf monkey, *T. obscurus* on the other hand indicate a clear leucism condition in which partial black skin coloration is present around the eyes, nose, lips, ears, both limbs and tail fur as shown in Fig. 2 and Table 2. Fig. 2 shows the comparison between normal morph and leucistic morph of *T. obscurus* based on face, ventral and side views. The face view (Fig 2-E1) of leucistic morph show a black skin patch remained around the nose and ears. The ventral view (Fig 2-E2) shows the hand and nipple are also black in color. The side views (Fig 2-E3) expose the feet and part of the tail is also has black fur.

Table 1 show the combination of keywords search for articles in SCOPUS database related to Colobinae, colobine, langurs, morph, leucism, albinism, colouration (coloration), pigmentation and all genus in colobine from subfamily Colobinae. Diversity of different morphs in colobines reported from previous studies were summarized in Table 2 including the red morph and black morph of *Trachypithecus cristatus* [6], the red morph and black morph of *Trachypithecus auratus* [7,8] and the white morph and black morph of *Semnopithecus vetulus vetulus* in Sri Lanka [9].

**Table 1:** Number of research articles in each bibliographic search related with unique colouration or morphism among colobines.

Search Topics	Total Number of Research Articles (% Already Included in Previous Research)	Suitable Peer-Reviewed Articles
Colobinae* AND albino*	0	-
Colobinae* AND albinism*	0	-
Colobinae* AND morph*	3	0
Colobinae* AND leucism*	0	-
Colobinae* AND coloration*	6	0
Colobinae* AND colouration*	0	-
Colobinae* AND pigmentation*	4 (50%)	0
colobines* AND albino*	0	-
colobines* AND albinism*	0	-
colobines* AND morph*	3 (66%)	0
colobines* AND leucism*	0	-
colobines* AND coloration*	0	-
colobines* AND colouration*	1	0
colobines* AND pigmentation*	2 (50%)	0
langur* AND albino*	2	0
langur* AND albinism*	0	-
langur* AND morph*	3 (66%)	1
langur* AND leucism*	0	-
langur* AND coloration*	3 (100%)	1
langur* AND colouration*	1 (100%)	0
langur* AND pigmentation*	2 (100%)	0
Presbytis* AND albino*	0	-
Presbytis* AND albinism*	0	-
Presbytis* AND morph*	1	0
Presbytis* AND leucism*	0	-
Presbytis* AND coloration*	3	0
Presbytis* AND colouration*	2 (50%)	0
Presbytis* AND pigmentation*	1 (100%)	0
Trachypithecus* AND albino*	1 (100%)	0
Trachypithecus* AND albinism*	0	-
Trachypithecus* AND morph*	3 (66%)	0
Trachypithecus* AND leucism*	0	-
Trachypithecus* AND coloration*	2 (50%)	0
Trachypithecus* AND colouration*	1 (100%)	0
Trachypithecus* AND pigmentation*	3 (100%)	0
Semnopithecus* AND albino*	0	-
Semnopithecus* AND albinism*	0	-
Semnopithecus* AND morph*	1 (100%)	1
Semnopithecus* AND leucism*	0	-
Semnopithecus* AND coloration*	1 (100%)	1
Semnopithecus* AND colouration*	0	-
Semnopithecus* AND pigmentation*	0	-
Rhinopithecus* AND albino*	0	-
Rhinopithecus* AND albinism*	0	-
Rhinopithecus* AND morph*	1 (100%)	0
Rhinopithecus* AND leucism*	0	-
Rhinopithecus* AND coloration*	3 (66%)	0
Rhinopithecus* AND colouration*	0	-
Rhinopithecus* AND pigmentation*	1 (100%)	0
Nasalis* AND albino*	1	0
Nasalis* AND albinism*	0	-
Nasalis* AND morph*	0	-
Nasalis* AND leucism*	0	-
Nasalis* AND coloration*	0	-
Nasalis* AND colouration*	0	-
Nasalis* AND pigmentation*	0	-
Pygathrix* AND albino*	0	-
Pygathrix* AND albinism*	0	-
Pygathrix* AND morph*	1 (100%)	0
Pygathrix* AND leucism*	0	-
Pygathrix* AND coloration*	2 (100%)	0
Pygathrix* AND colouration*	0	-
Pygathrix* AND pigmentation*	1 (100%)	0
Colobus* AND albino*	1	1
Colobus* AND albinism*	0	-
Colobus* AND morph*	1 (100%)	0
Colobus* AND leucism*	0	-
Colobus* AND coloration*	3 (66%)	0
Colobus* AND colouration*	0	-
Colobus* AND pigmentation*	0	-
Piliocolobus* AND albino*	0	-
Piliocolobus* AND albinism*	0	-
Piliocolobus* AND morph*	1 (100%)	0
Piliocolobus* AND leucism*	0	-
Piliocolobus* AND coloration*	0	-
Piliocolobus* AND colouration*	0	-
Piliocolobus* AND pigmentation*	0	-
Procolobus* AND albino*	0	-
Procolobus* AND albinism*	0	-
Procolobus* AND morph*	1 (100%)	0
Procolobus* AND leucism*	0	-
Procolobus* AND coloration*	0	-
Procolobus* AND colouration*	1 (100%)	0
Procolobus* AND pigmentation*	0	-
**Total number of research articles**	67	5

**Table 2:** The review of different morph reported in different species of colobine.

Species	Normal morph	Others morph	Type of morph	Gender of the other morph	Age group	Reference & location
*Presbytis femoralis*			Brown morph	Male	Adult	This report Kampung Johor Lama, Kota Tinggi, Johor, Malaysia
*Trachypithecus obscurus*			White morph (leucism)	Female	Adult	This report Langkawi, Kedah, Malaysia
*Trachypithecus cristatus*			Red morph	Undetermined	Sub-adult	[6] Sabah, Malaysia
*Trachypithecus auratus*			Red morph	Female	Adult	[7,8]
*Semnopithecus vetulus vetulus*			leucistic	Undetermined	Adult	[9] Matara, Sri Lanka

## 2 Experimental Design, Materials, and Methods

The observation was conducted in Johor and Langkawi, Malaysia. *P. femoralis*, were observed in Kampung Johor Lama, Kota Tinggi, Johor (1.585043°N, 104.014104°E). The focal group, named “Dara,” was an all-male group of banded langurs which consisted of four identified members (Ireng, Dara, Aswad, and Puteh). Observations began in February 2018 until November 2018 started at 7:00 AM to 7:00 PM every sampling day [10]. The observations on brown morph in banded langurs presented here were recorded as part of the ethological observation on *P. femoralis* in Johor, Malaysia [11,12]. Observation was aided by Canon 60D digital single lens reflex (DSLR) camera with Canon 70-300mm F4-5.6L lens for taking photos of brown morph individual's face, side and ventral view to differentiate its morphology.

Another data collection is on leucistic dusky leaf monkey in Burau Bay, Langkawi, Kedah Malaysia (6.366938 °N, 99.667521°E). We discovered this individual during DNA data collection on primates of Malaysia focusing on slow loris, *Nycticebus coucang* in Langkawi Island [13]. The discovery of leucistic individual was accidental and unplanned thus, the data available here is limited.

Bibliographic searches were used to obtain data on diversity of morph reported among Colobines. Peer-reviewed articles were searched in SCOPUS database, according to indexed title, abstract, keywords and topics and by using a few search strings listed in the Table 1 above. In total, 35 out of 84 search strings listed were successfully exported as the others yielded no data (Table 1). Thus, 67 peer-reviewed articles were considered in our review. From 67 articles, the search strings were further filtered to see if the articles provide and discuss information regarding morphism or leucism, among which five were deemed acceptable. We defined ‘acceptable’ here as any articles that focus on and contains actual report and photograph of unique coat coloration or morphism among colobines. However, the five search string results that were considered acceptable refers to only two articles [9,14]. However only one [9] article was included in Table 2 as the other article [14] did not provide photographic evidence.

Comparing with other morphs of *T. cristatus, S. v. vetulus*, and *T. auratus*, the brown morph of *P. femoralis* is another sporadic phenomenon to be reported. The occurrence of brown morph may be because of absence of eumelanin expression (black/brown) and pheomelanin (red/yellow) are solely expressed in the hair shaft [5]. Thus, the hair was expressed as brown fur instead of black. The case of red-orange *T. cristatus* found in the Kinabatangan River, Sabah, expressed the same hair coloration as the infant of the species [6]. *T. auratus* also exhibits a red, “erythristic” morph, which occurs within a restricted area of eastern Java between Blitar, Ijen, and Pugeran [8,15]. Leucistic morph of *T. obscurus* describe in this report was almost similar to the case of *S. v. vetulus* in Sri Lanka [9]*.* The case of *S. v. vetulus* in Sri Lanka shown that the morph is white and has black patches which can be classified as leucistic. It was reported up to 30 individuals in 26 troops found in south Sri Lanka making it a much more common morph than those found in other species [9]. The current report on the brown morph of *P. femoralis* in Johor and leucistic morph of *T. obscurus* need further research from molecular and ecological perspectives.

*P. femoralis* and *T. obscurus* individuals showed no distinct behavior among individuals within the group as per our observation. The morphology of both langurs did not show any indication of albinism, such as total absence of integumentary and retinal pigmentation [2]. The closer term for the case of *T. obscurus* would be leucism, in which there are pigmented retinas but reduced or absent integumentary pigment [3,4].

## Declaration of Competing Interest

The authors declare that they have no known competing financial interests or personal relationships which have, or could be perceived to have, influenced the work reported in this article.

## Appendix Supplementary materials

Image, application 1

Image, application 2
