# Supplementary material for: Data on First Record of Brown Morph Banded Langur (Presbytis femoralis), Leucistic Dusky Leaf Monkey (Trachypithecus obscurus) in Malaysia and Review of Morph Diversity in Langur (Colobinae)
Source: Data Brief. 2020 May 21;31:105727. doi: 10.1016/j.dib.2020.105727 (PMC7284059; doi:10.1016/j.dib.2020.105727)
Supplement: Supplementary file 1 [file mmc1.docx]

Supplementary File - Photos of normal morph and brown morph of *P. femoralis*
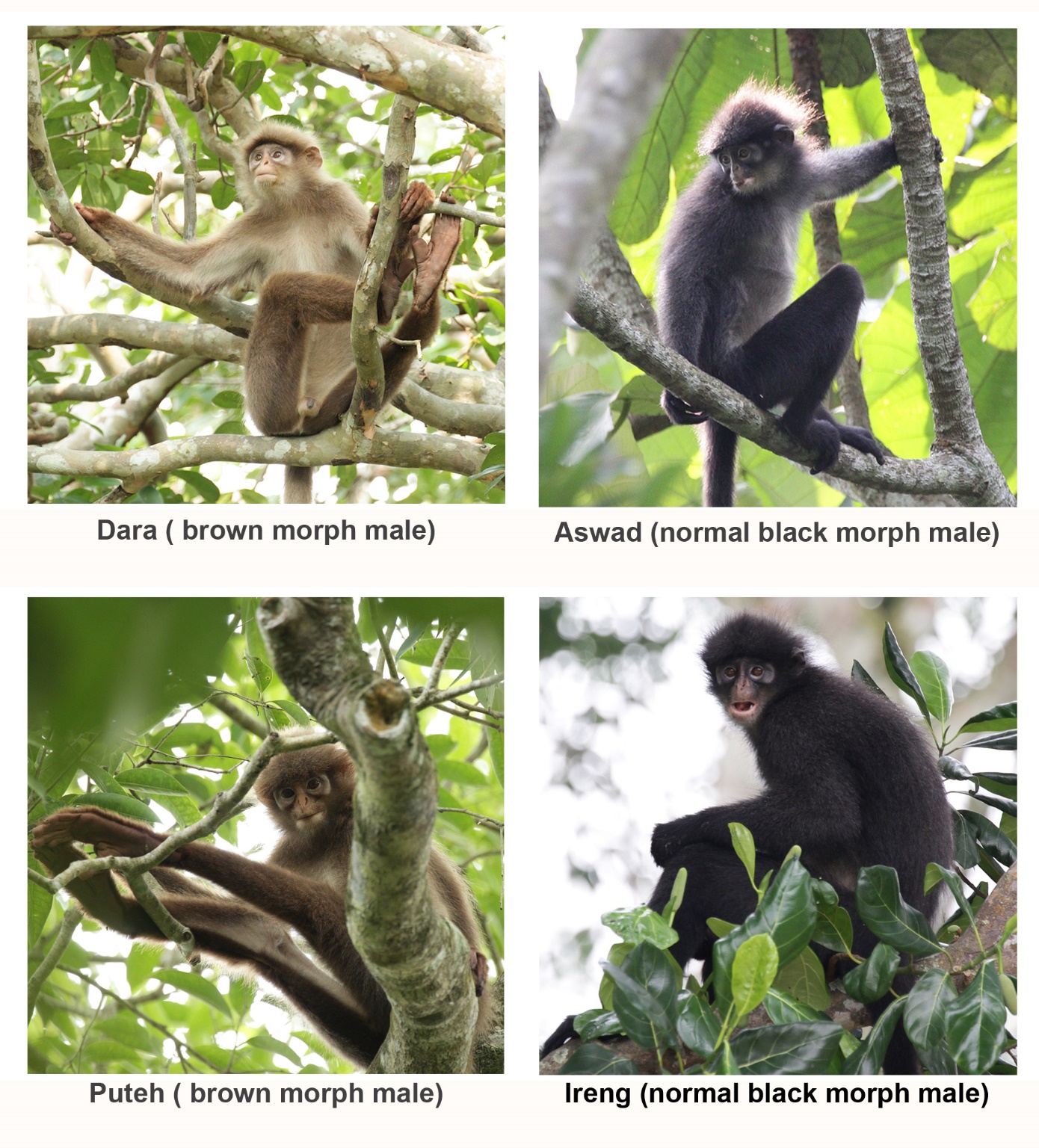


S1. Photo collage of all individuals in focal group of our study for *P. femoralis* in Kampung Johor Lama, Kota Tinggi, Johor.


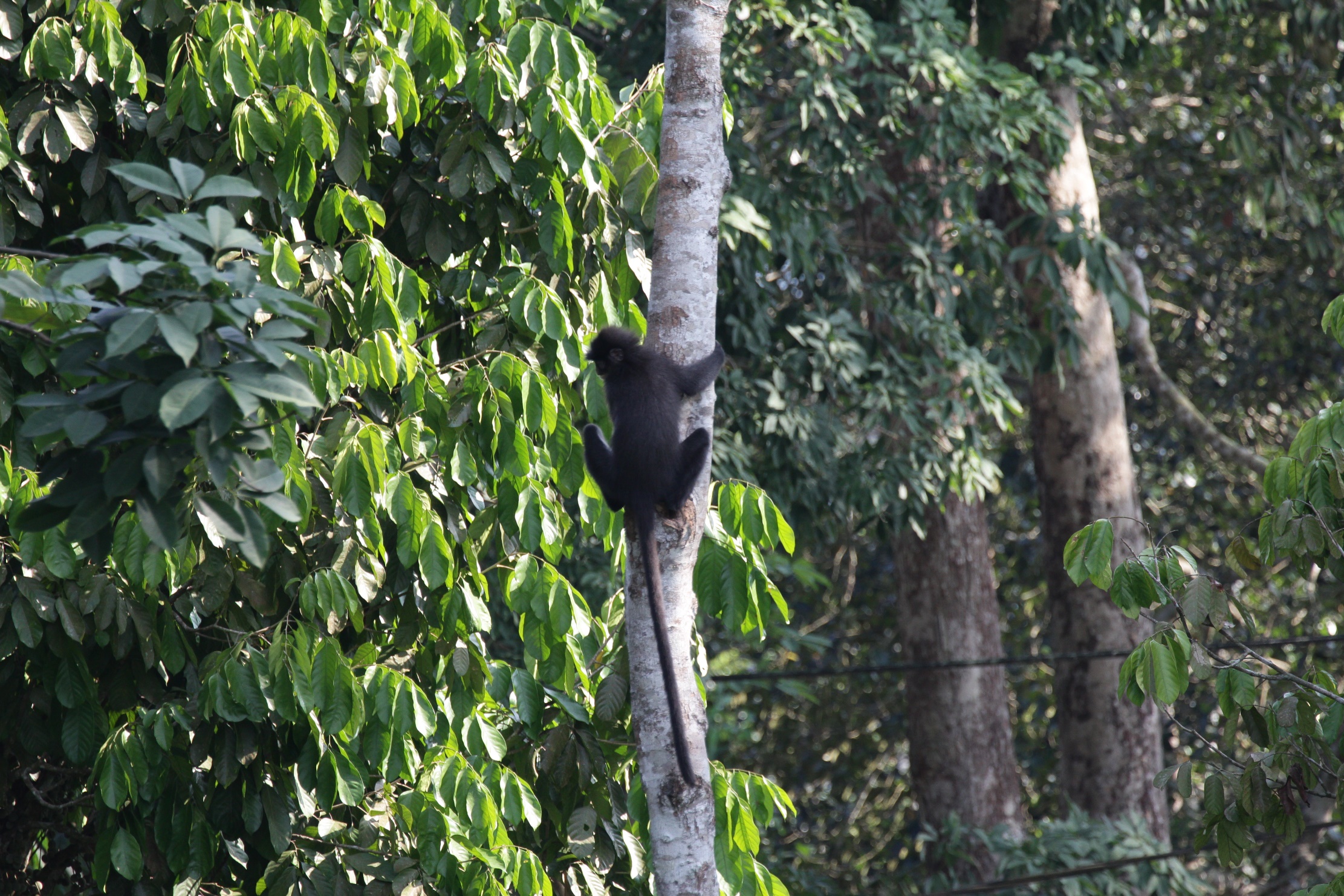

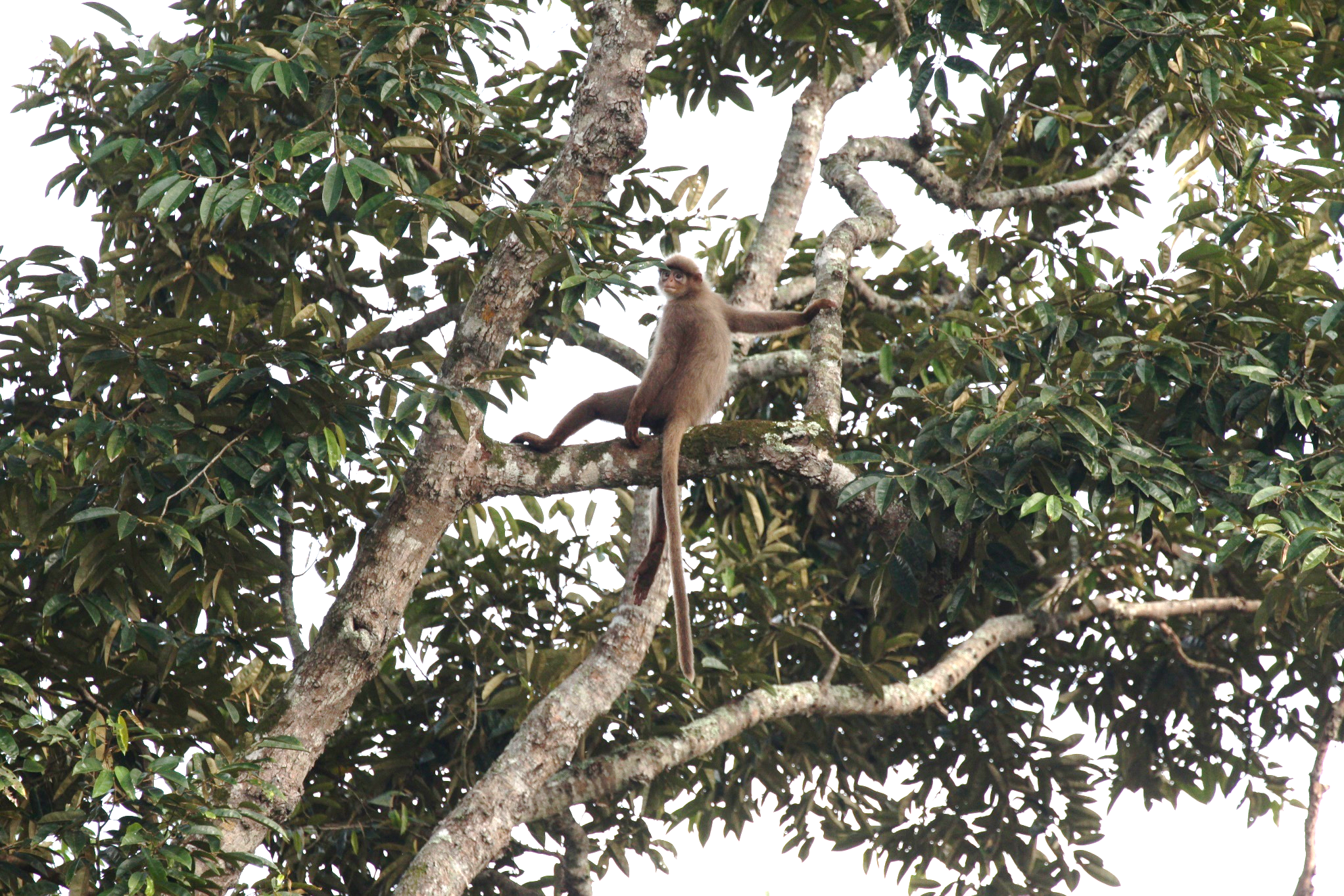


S2. The comparison between dorsal torso and tail of Ireng (normal black morph male) and Dara (brown morph male).


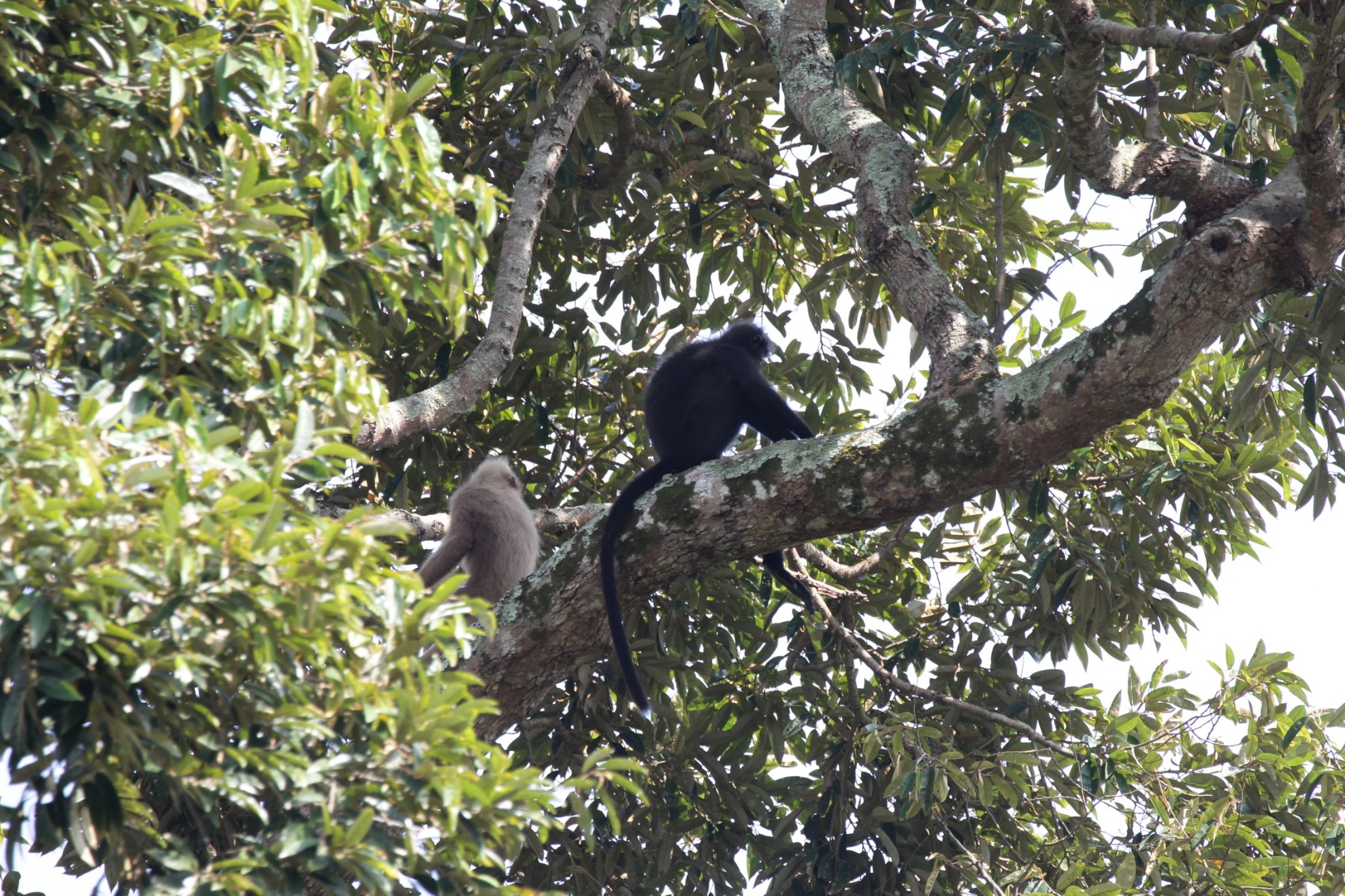


S3. The comparison between dorsal torso of Puteh (brown morph male) and Aswad (normal black morph)


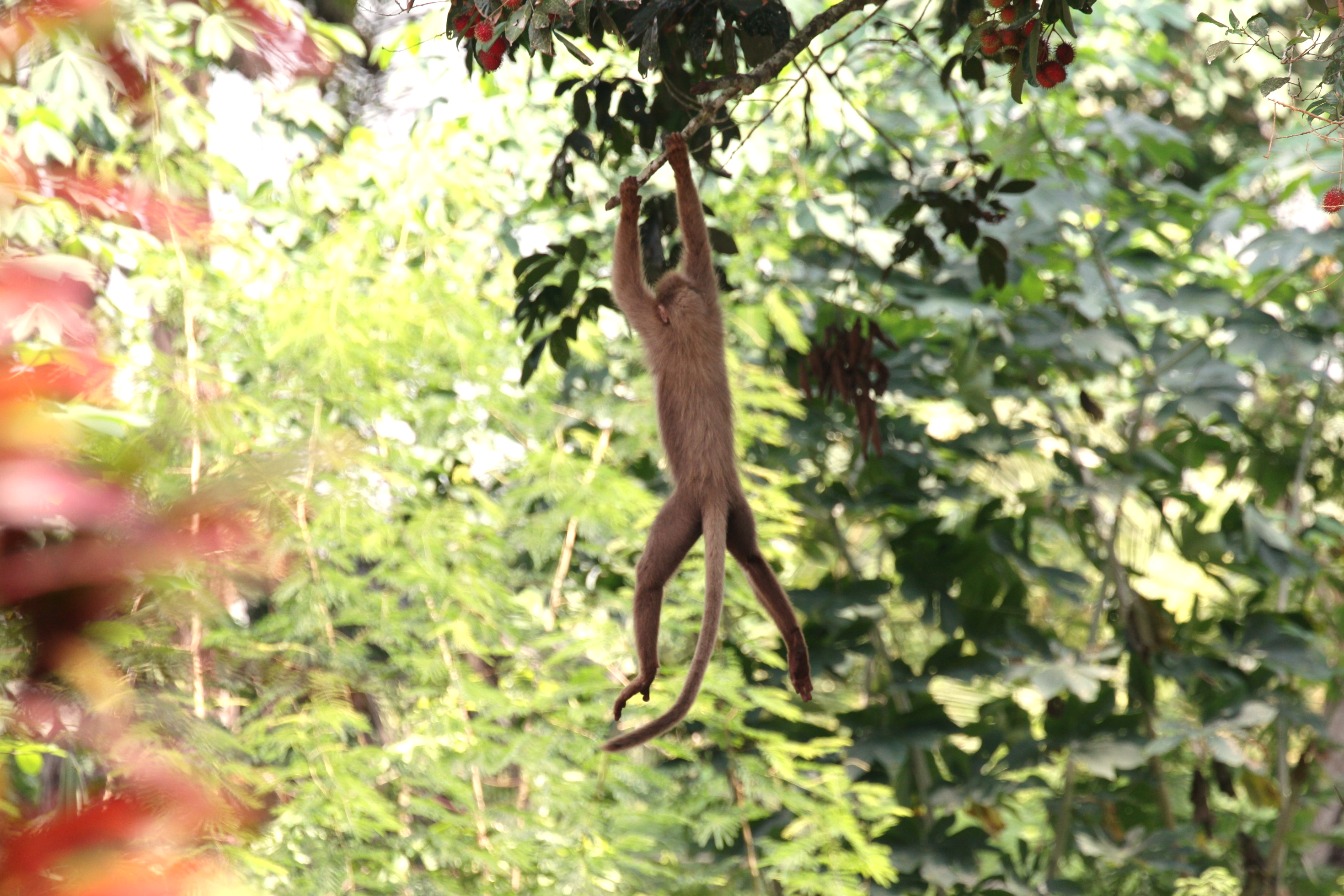


S4. Full dorsal torso and tail of Dara (brown morph male) of *P. femoralis.*


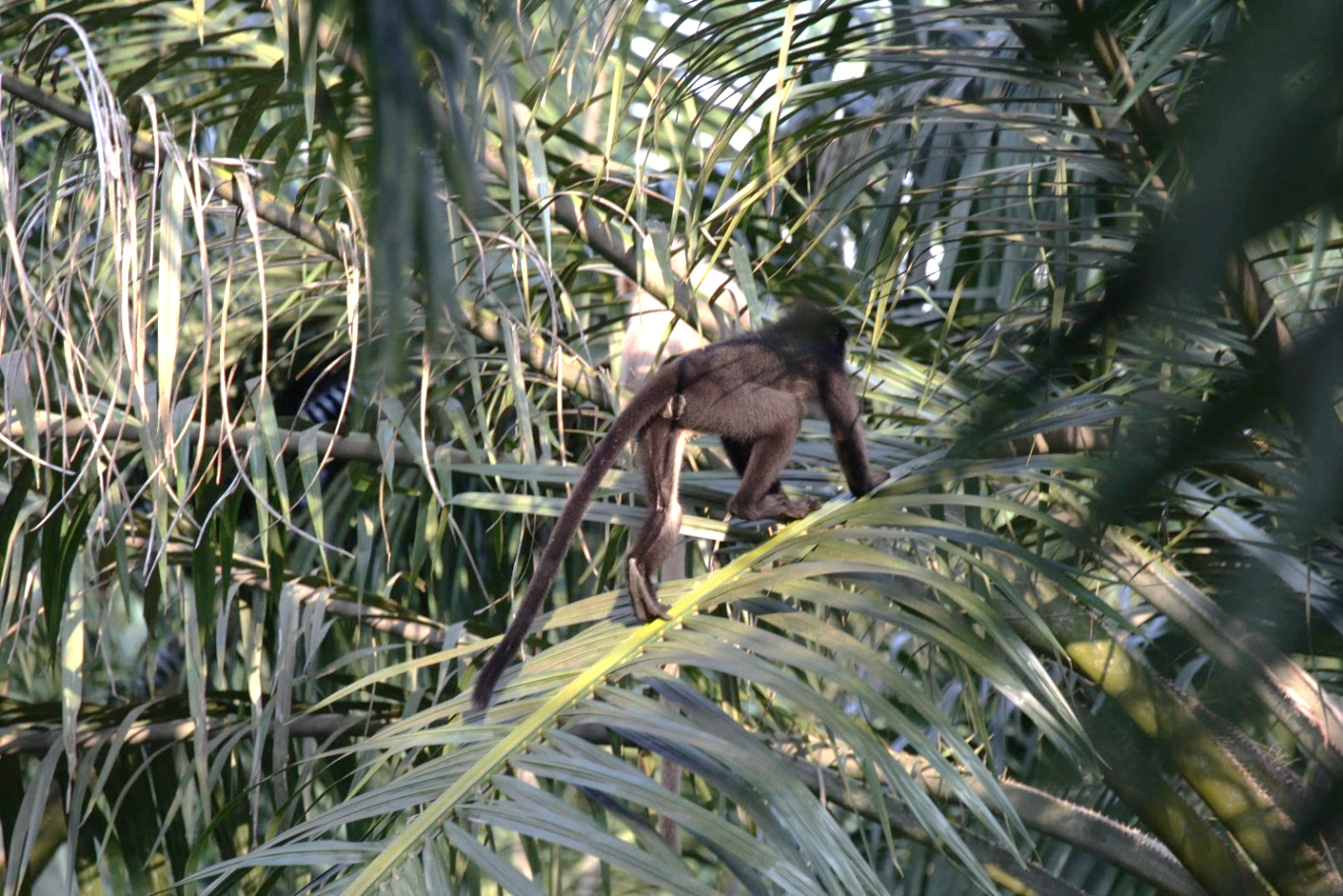


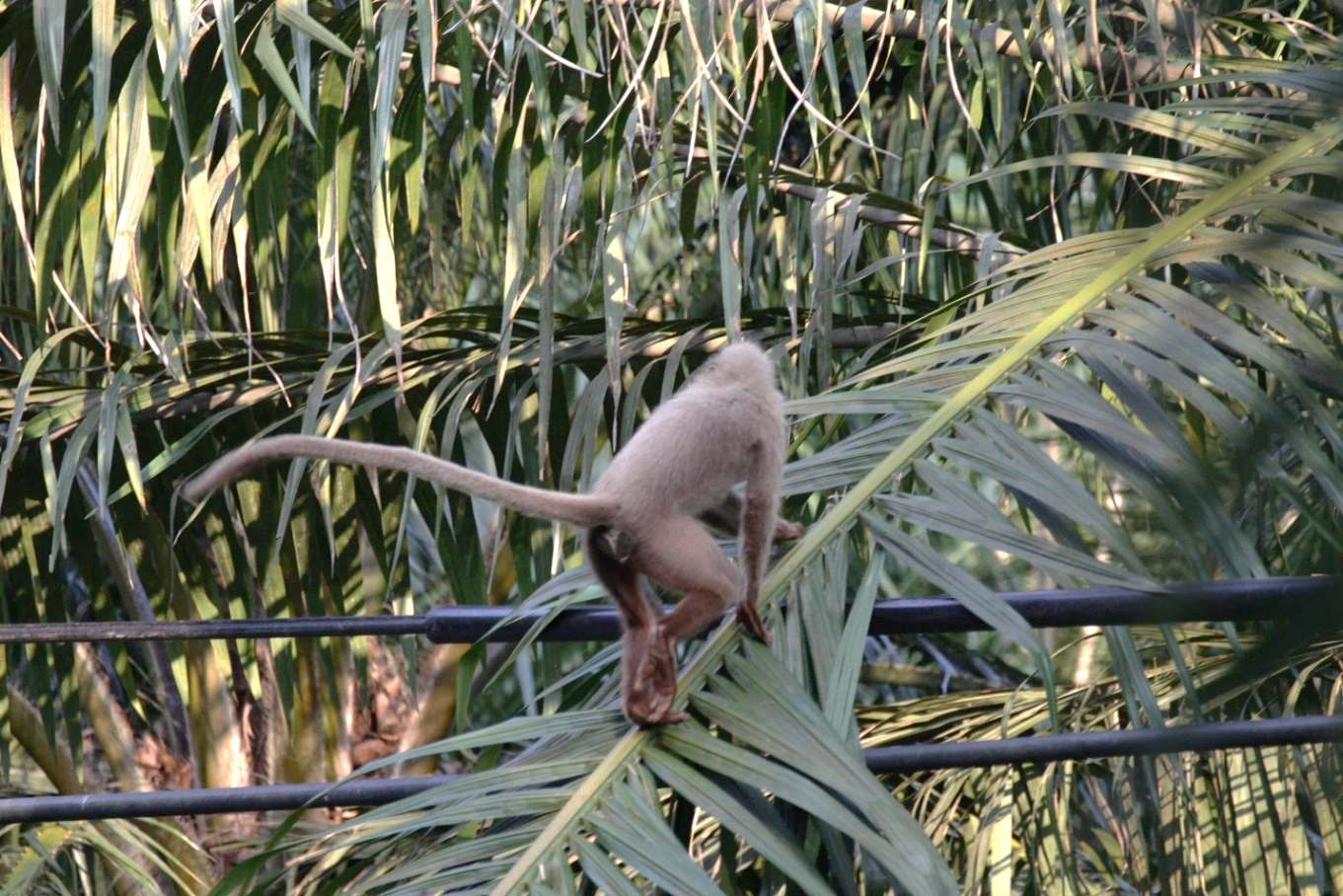


S5. The comparison of rear view of Aswad (normal black morph male) and Dara (brown morph male) of *P. femoralis*.


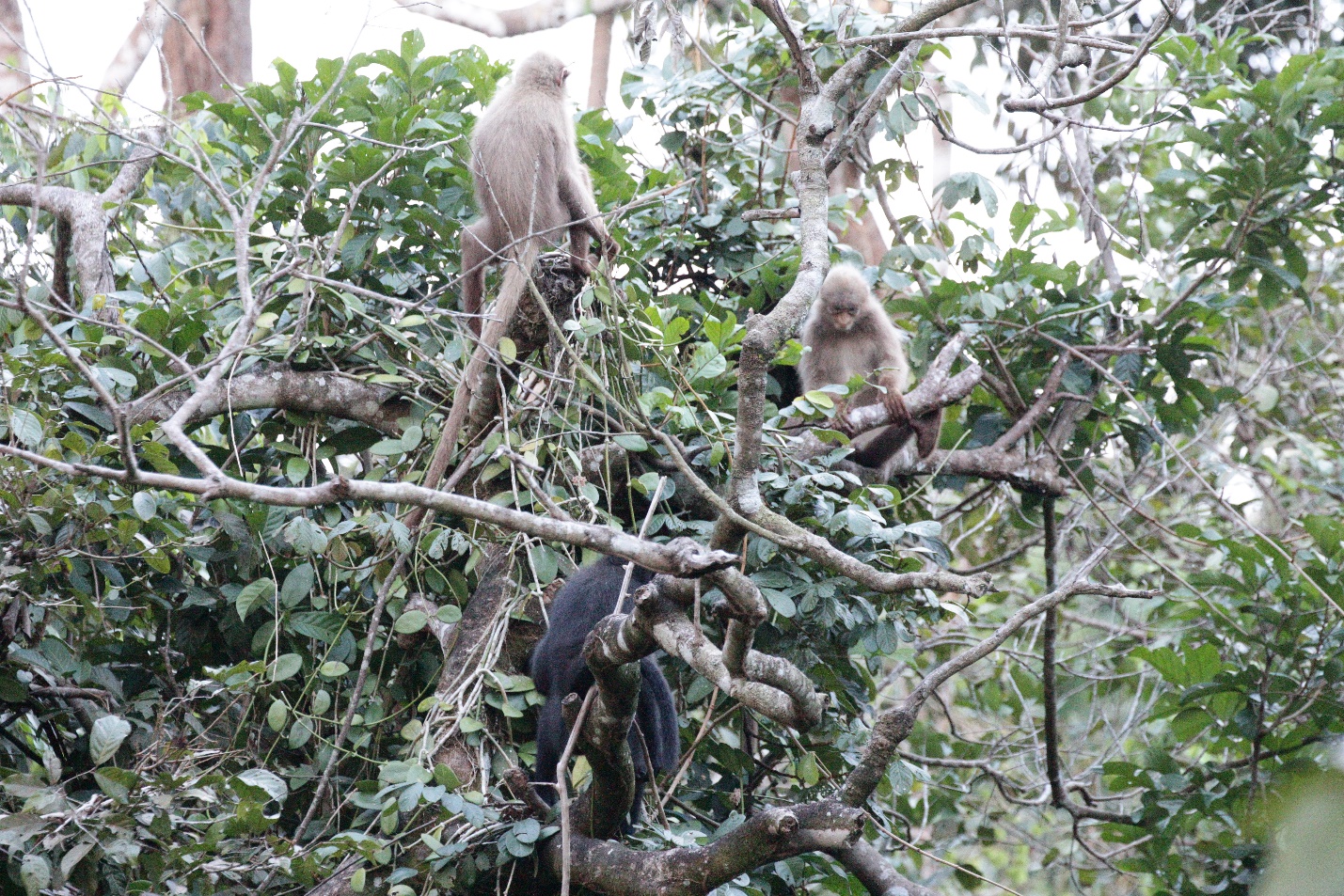


S6. Feeding behaviour of Puteh (brown male morph), Dara (brown male morph) and Aswad (black male morph).


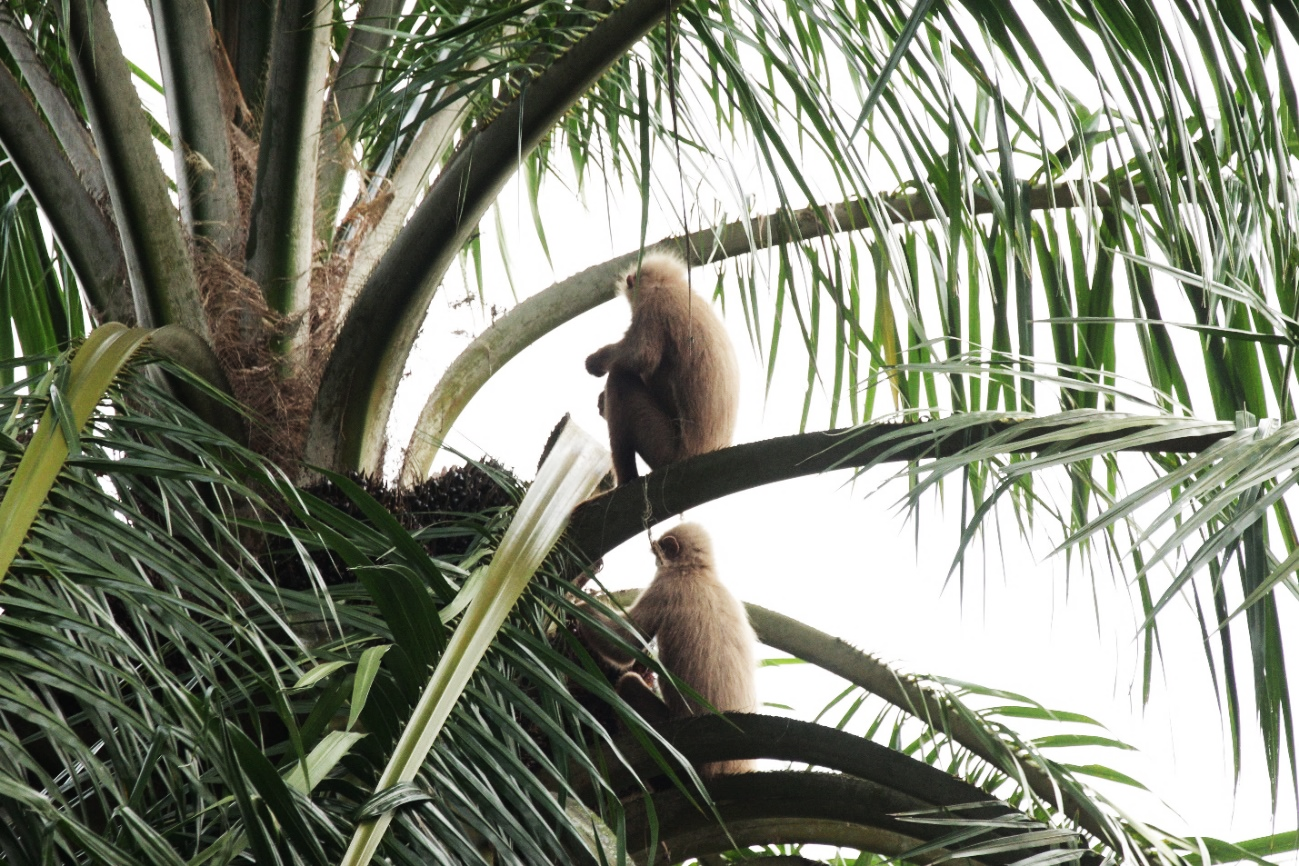


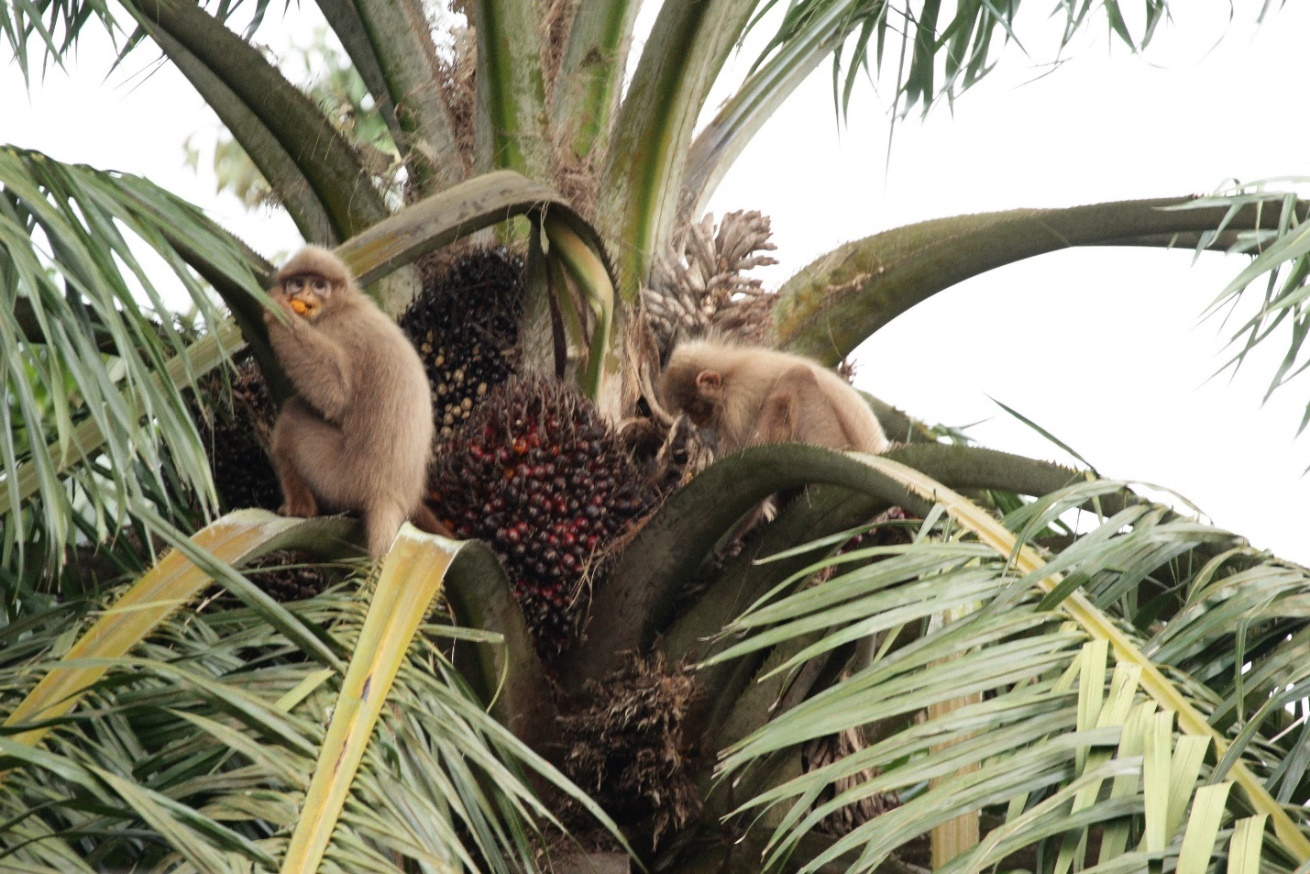


S7. Feeding behaviour of two brown morph of *P. femoralis* on palm oil tree (*Elaeis guineensis*).


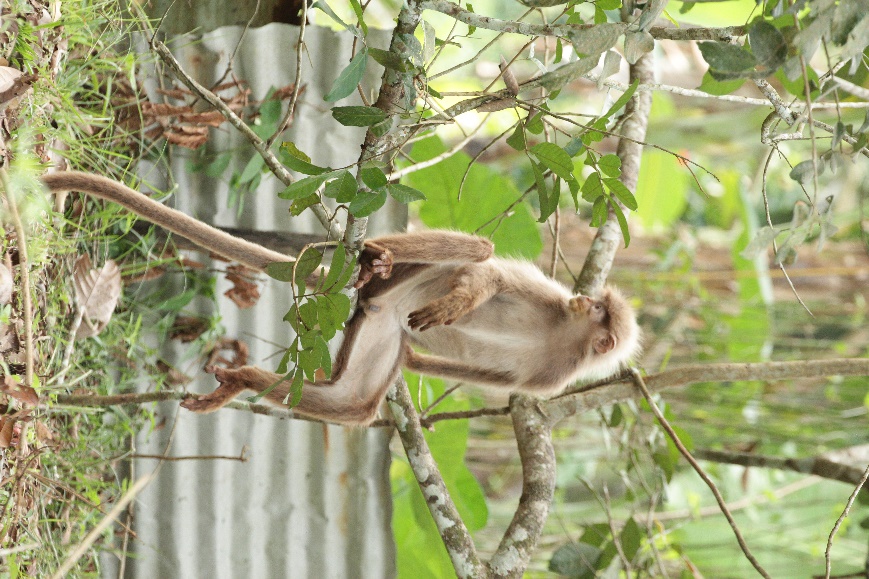

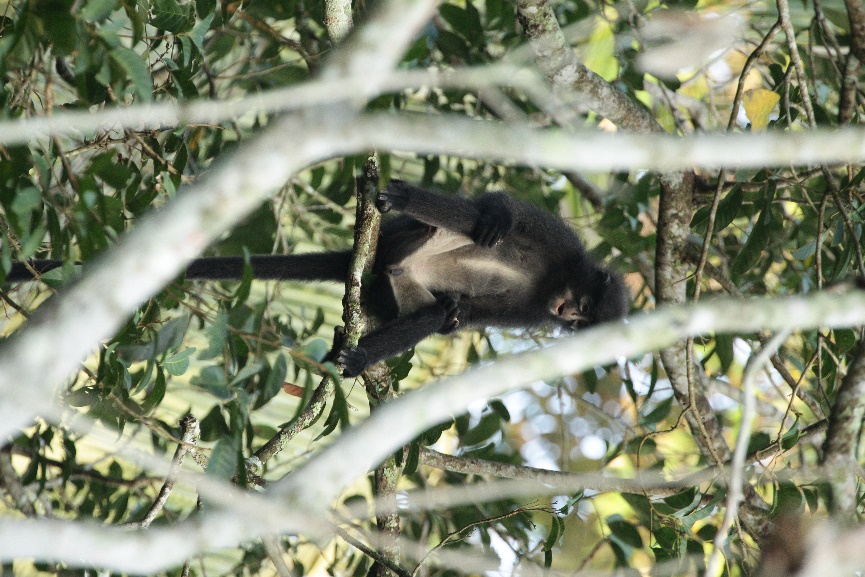


S8. Comparison of ventral body area between Puteh (brown morph) and Ireng (black morph).
